# Time-series analysis of presentations to four syringe dispensing machines and a needle and syringe programme during COVID-19 lockdowns in Melbourne, Australia

**DOI:** 10.1186/s12954-022-00683-9

**Published:** 2022-09-07

**Authors:** Daniel O’Keefe, Michael Livingston, Reece D. Cossar, Phoebe Kerr, David Jacka, Paul Dietze

**Affiliations:** 1 Behaviours and Health Risks Program, Burnet Institute, 85 Commercial Road, VIC 3004 Melbourne, Australia; 2 School of Public Health and Preventive Medicine, Monash University, Melbourne, VIC Australia; 3 Faculty of Health Sciences, National Drug Research Institute and enAble Institute, Curtin University, Perth, WA Australia; 4 Centre for Alcohol Policy Research, La Trobe University, Melbourne, VIC Australia; 5 Drug and Alcohol Service, Monash Health, Dandenong, VIC Australia

**Keywords:** Syringe-dispensing machine, Syringe vending machine, Needle and syringe programme, Harm reduction

## Abstract

**Background:**

Australian harm reduction services are provided via a mix of modalities, including fixed-site needle and syringe programmes (NSP) and syringe-dispensing machines (SDMs). SDMs are cost-effective and provide 24-h anonymous access to needles/syringes, often to underserved geographic areas, and can attract clientele who may choose not to use NSPs. The introduction of COVID-19 control measures saw disruptions and adaptations to the provision of harm reduction services. It is possible that SDMs filled the gap in otherwise disrupted harm reduction services in Melbourne. In this paper, we use data from four SDMs and an NSP to explore changes to harm reduction usage during periods of COVID-19 lockdowns in Melbourne, Australia, in 2020.

**Methods:**

Our data span September 2017–December 2020. We analysed daily counts of SDM use and monthly counts of NSP use, according to unique presentations to both. Auto-regressive integrated moving average (ARIMA) time-series models were fitted to the data with the effects of lockdowns estimated via a step function.

**Results:**

Across the study period, we estimated 85,851 SDM presentations and 29,051 NSP presentations. Usage across both the SDMs and the NSP declined during the COVID-19 lockdowns, but only the decline in SDM usage was significant in ARIMA analysis.

**Conclusions:**

The slight, but significant decline in SDM use suggests barriers to access, though this may have been mitigated by SDM users acquiring needles/syringes from other sources. The decline, however, may be a concern if it led to lowered needle/syringe coverage and a subsequent increase in injecting risk. Further work is needed to properly explore potential changes in preference for needle/syringe acquisition site and associated barriers. Importantly, this work adds to the body of literature around the impacts of COVID-19 on harm reduction provision and potential areas of improvement.

## Background

Australia has high population-level needle/syringe distribution coverage [1, 2], which is achieved through free distribution with few restrictions on the number of needles/syringes an individual may acquire during any one presentation [2]. Further, harm reduction access is enhanced by distributing needles/syringes via a mixture of modalities, including primary needle and syringe programmes (NSPs) (specific harm reduction services targeting people who inject drugs, often with on-site ancillary services), secondary NSPs (NSPs operating from non-targeted sites, such as community health centres and hospitals), pharmacies, peer-to-peer distribution, and mobile outreach services (both on foot and by vehicle) [3, 4]. These services often operate synergistically. For example, a primary fixed-site NSP may also operate a peer-to-peer distribution programme and a mobile outreach service to increase coverage, particularly during times when the fixed-site NSP is closed. In total, it is estimated Australia distributed 50.2 million needles/syringes in the 2020/21 financial year via 4218 outlets to an estimated 74,000 people who inject drugs, or approximately 675 needles/syringes distributed per person per annum [5]. Australia’s early adoption of harm reduction interventions, comprehensive implementation of varied modalities, and high needle/syringe coverage levels are often cited as central to averting an HIV epidemic among people who inject drugs, as experienced in other countries [3].

Syringe-dispensing machines (SDMs), which provide sterile injecting equipment via an anonymous vending machine, are also used in Australia [5]. There were an estimated 399 SDMs operating nationally in 2021 [5], generally managed by existing harm reduction services and often located directly outside fixed-site services. SDMs are cost-effective and provide 24-h access to needles/syringes, often to underserved geographic areas, and can attract clientele who may choose not to use fixed-site NSPs [6–9]. The characteristics of people who frequent NSPs are thought to differ from people who use other forms of needle/syringe distribution [10]. Non-, or irregular, attendees of NSPs in Sydney, Australia, were less likely to have severe substance use dependence, a lower prevalence of blood-borne virus infection but lower rates of testing [10]. Importantly, people who use SDMs may desire greater anonymity or disassociation with the wider community of people who inject drugs [9], meaning that SDMs play a vital role in providing harm reduction access to a diverse range of people who inject drugs.

Both in Australia and internationally, the introduction of control measures due to the COVID-19 pandemic saw disruptions and adaptations to the provision of NSPs, such as disruptions to supply chains, closure of fixed-site walk-in services, changes in service hours and reduced staff capacity, that continue to affect NSPs [5, 11, 12]. Melbourne, Australia, experienced some of the most comprehensive and strict COVID-19 control measures compared to its international counterparts, including stay-at-home curfews, interstate travel bans, severe limits on social gatherings, mandated mask-wearing, and multiple, extended periods of city-wide lockdowns [13]. To mitigate negative consequences, NSPs actively encouraged clients to procure sufficient needles/syringes to last during COVID-19 lockdowns [5]. These changes and disruptions to normal needle/syringe distribution emphasised the importance of alternate and varied service modalities, such as SDMs, to adequately service all clients accessing harm reduction services [11, 12]. Contactless access to NSPs is a novel way to ensure that services remain available to people despite reduced staffing or during periods of restrictions on movement and gathering. However, while the procurement of sterile injecting equipment was considered an “essential service” during Melbourne lockdowns, and therefore a permitted reason to be outside the home, this activity also increased the likelihood of involvement with law enforcement as police sought to enforce social distancing regulations [14]. This potentially imposed a barrier to accessing even an available source of needle/syringes, such as SDMs. Otiashvili et al. [6] described a “remarkable increase” in the use of 10 SDMs during COVID-19 lockdowns in Tbilisi, Georgia. Data from Sydney, however, indicated no substantial change in SDM access, but also that 15% of participants reported trouble accessing needles/syringes from their usual source, principally due to SDMs being out of stock [11]. It is unclear whether SDMs filled the gap in otherwise disrupted harm reduction services in Melbourne.

In this paper, we explore changes in SDM and NSP usage during periods of government-implemented COVID-19 lockdowns in Melbourne, Australia, in 2020. Specifically, we use data from four SDMs, installed with innovative data capture systems, in south-eastern Melbourne and client-level presentation data from the NSP that manages the SDMs. We analysed trends in SDM and NSP presentations during periods of comprehensive COVID-19 lockdown to assess the resulting differences in utilisation. We hypothesise an increase in SDM use during the lockdown periods, with this work providing indication of the effectiveness of SDMs to meet challenges in fixed-site NSP provision during times of disruption, such as COVID-19.

## Methods

### Design and setting

In 2014, a primary fixed-site NSP (Monash Community NSP, MCNSP) in the south-eastern Melbourne suburb of Dandenong established four SDMs, located in multiple areas of south-east Melbourne (Dandenong, Berwick, Pakenham and Clayton). The MCNSP and four SDMs are all located in the public health catchment area of Monash Health. We selected these SDMs for analysis due to their data capture capability (described below) and the ability to compare presentation data against the managing MCNSP.

The four SDMs distribute a mix of cost (AUD$2) and free packs of sterile injecting equipment, including between six and eight needles/syringes (depending on pack type), swabs, a plastic spoon, one condom and water-based lubricant, one cotton wool filter and one health promotion leaflet.

### Data used and data preparation

The SDMs automatically log data on the pack-type dispensed and the time/day of the dispensation. Victorian NSPs are required to record data on client presentations for reporting purposes via the Needle and Syringe Program Information System (NSPIS) [15]. NSPIS data were provided as monthly aggregates of total client presentations, while the SDM data logs every order made. The span of SDM and NSP comparative data was September 2017–December 2020 (40 months). Both SDM and NSP data were provided by the MCNSP.

Because an individual can make multiple SDM orders during a single presentation, and to equate SDM and NSP presentation data, it was necessary to estimate SDM access by ‘unique’ individuals or groups of individuals (irrespective of how many orders were placed during a single presentation). We estimated unique presentations by applying a 45-s cut-off between SDM orders. We presumed that a unique person could make a single SDM order during the same presentation within 45 s per order. Therefore, any number of SDM orders could be made during a unique presentation, so long as no single order was made 45 s after the last sequential order. Any order occurring more than 45 s since the previous order was assumed to be a different unique presentation.

### Data analysis

We provide a descriptive analysis of SDM and MCNSP presentations during the analysis period.

To assess the impacts on service access due to COVID-related restrictions in Melbourne during 2020 (city-wide lockdowns occurred between April 1–May 12 and July 9–October 27 [16]), we analysed daily counts of unique SDM presentations and monthly counts of unique MCNSP presentations. Auto-regressive integrated moving average (ARIMA) time-series models were fitted to the data, with categorical variables for the month of year and day of week included to model seasonality and weekly periodicity in SDM use. The effects of lockdowns were estimated both via a step function (0 when the lockdown was not in place, 1 when it was) and a slope function (a simple count variable increasing from 1 on the first day of a lockdown to *n* on the nth day, and then resetting to 0 when the lockdown was lifted). This allowed us to assess whether lockdowns led to an immediate impact on SDM use (via the step function) or whether use shifted over the course of a lockdown (via the slope function). ARIMA models were fitted iteratively, starting with the most basic model (all ARIMA parameters = 0) and increasing in complexity until residuals approximated white noise (checked via Auto Correlation Function plots and the Portmanteau Q test).

Due to the level of aggregation in MCNSP data, we only modelled lockdown as a step function for these data, with each month given a value between 0 (no lockdown) and 1 (lockdown spanning the full month) depending on the proportion of the month Melbourne spent in lockdown. Seasonal effects were adjusted by including month as a categorical variable.

## Results

### Descriptive analysis of SDM and NSP usage

Across the analysis period, 172,205 total orders were made across the four SDMs (Berwick = 16,139 (9.37%); Dandenong = 108,394 (62.94%); Clayton = 27,821 (16.16%); and Pakenham = 19,851 (11.53%)). A total of 1,298,553 needles/syringes were dispensed via the SDMs. During the same period, a total of 1,717,800 needles/syringes were distributed through MCNSP over 29,051 unique presentations.

We estimated 85,851 ‘unique’ SDM presentations. The distribution of unique presentations across the four SDMs was relatively consistent with total orders: Berwick = 9079 (10.58%); Dandenong = 53,981 (62.88%); Clayton = 14,023 (16.33%); and Pakenham = 8768 (10.21%). The median number of SDM orders during unique presentations was one (IQR 1–2, range 1–120; the 120 attempts due to repeated attempts with the SDM being sold out of product). The median number of needles/syringes successfully dispensed during unique presentations was 8 (IQR 8–16, range 1–552). There was no difference in the median number of unique presentations during the lockdown periods and non-lockdown periods or the number of needles/syringes successfully dispensed. During the analysis period, 31.64% of total unique SDM presentations (*n* = 27,166) occurred during MCNSP opening hours (Monday-Friday, 9am–5 pm), while during the first lockdown period, 34.64% (*n* = 1119) occurred during MCNSP opening hours and 38.05% (*n* = 2906) of all unique presentations during the second lockdown period.

We were unable to assess changes in average MCNSP presentations or distributed needles/syringes across the lockdown periods due to the MCNSP data being provided in monthly aggregates, and the lockdown start/stop dates occurring mid-month.

### Interrupted time-series analysis of SDM and NSP presentations

**Fig. 1:**
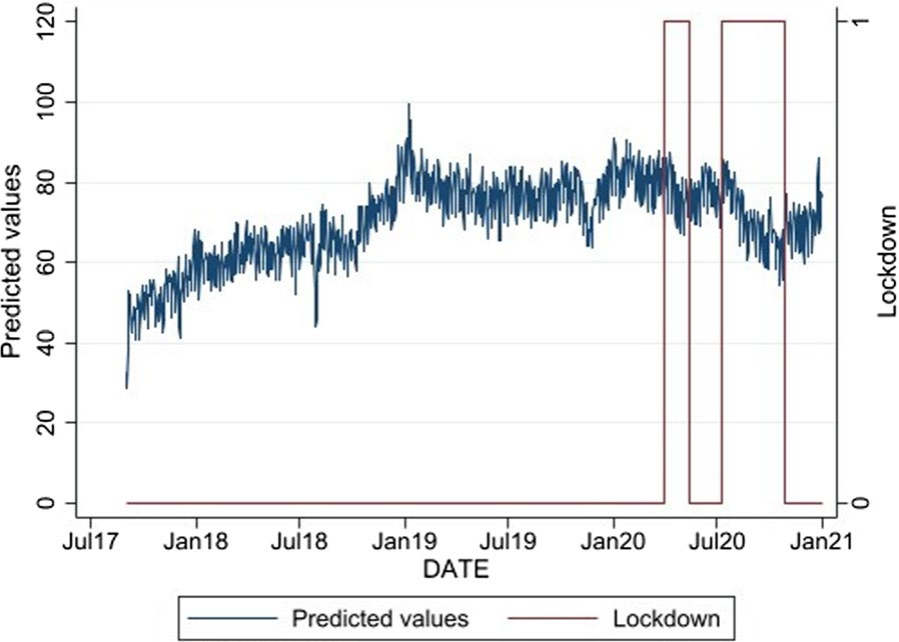
Predicted values of unique SDM presentations, September 2017–December 2020

**Fig. 2:**
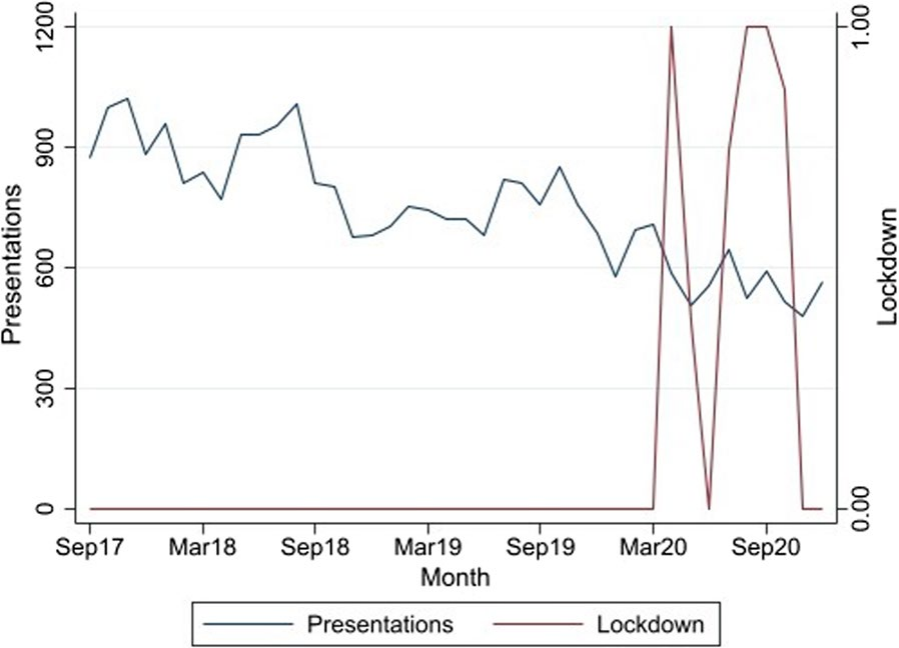
Monthly attendances at NSPs in Dandenong, September 2017–December 2020

The trend of daily unique presentations to the SDM between September 2017 and December 2020 is presented in Fig. 1, with the periods of COVID-19 lockdown indicated. Similarly, the monthly attendances at MCNSP are presented in Fig. 2.

Time-series models assessing the impact of lockdowns on needle/syringe distribution are presented in Table 1. The SDM models incorporate both step and trend parameters for the lockdown period because daily data were available, while the MCNSP model examined only a step function.

**Table 1:** ARIMA models assessing the impact of lockdowns on the use of SDMs and on MCNSP presentations

	Estimate	*p* value	95% CI
*SDM model estimates*^a^			
Lockdown step	3.85	0.29	− 3.22, 10.92
Lockdown trend	− 0.12	0.03	− 0.23, − 0.01
*MCNSP model estimates*^b^			
Lockdown step	− 32.1	0.71	− 200.0, 135.8

SDM model adjusted for month of year and day of week, MCNSP model for month of year

^a^*N* = 1217, ARIMA(2,1,2), Portmanteau Q test for residuals: *p* = 0.475

^b^*N* = 39, ARIMA(1,1,0), Portmanteau Q test for residuals: *p* = 0.408

In the SDM model, there was a significant negative slope effect for the lockdowns, which means that SDM unique presentations decreased steadily across the lockdown periods. The raw MCNSP presentation trend data showed a steady decline in MCNSP unique presentations during the study period, but that there was no significant relationship observed between MCNSP presentations and the periods of Melbourne lockdowns in the ARIMA models.

## Discussion

This paper describes the trends in utilisation of four SDMs and a fixed-site primary NSP located in South-East Melbourne, Australia, during COVID-19 lockdown periods in 2020. Time-series analysis showed a significant decline in SDM unique presentations during the two lockdowns, but no significant change in MCNSP fixed-site presentations. Our findings contrast with those of Otiashvili et al. [6], who described substantial increases in SDM use in Georgia, noting the increase coincided with access barriers to NSPs, such as lack of public transportation and limited opening hours.

The 2020 Illicit Drug Reporting System (IDRS), an annual, national surveillance survey of Australian people who inject drugs, included questions about changes to drug use and service access due to COVID-19. Forty-eight per cent and 36% of the sample reported a decline or cessation in methamphetamine and heroin use, respectively (either injecting or non-injecting use), principally due to ‘decreased availability’ of the drug [17]. Twelve per cent of the national sample also reported difficulty in obtaining needles/syringes from any source since March 2020 [17] (IDRS recruitment occurred between June and September 2020, as such, some interviews may not have covered lockdown periods analysed). Similarly, very few participants reported increases in receptive needle/syringe sharing or syringe reuse [17]. Reductions in drug injection surely had an impact on service use, but importantly, the IDRS results suggest that despite changes in the delivery of NSP services, people who inject drugs were still largely able to acquire sufficient sterile needles/syringes during 2020. However, the IDRS survey recruits many of its participants from NSPs and may not sample people who prefer to acquire needles/syringes from SDMs. Further, the 2020 iteration of the IDRS survey required an amended methodology to operate within relevant COVID-19 restrictions, meaning there may have been differences in sampled participants compared to other years [17]. Consequently, IDRS results may not reflect people who regularly use the SDMs, or people who chose not to attend NSPs. For some, the Melbourne lockdowns may have meant SDM access was less preferable to attending the NSP, where a much larger number of sterile needles/syringes can be acquired and potentially stockpiled during the uncertainty of the COVID-19 lockdowns. This practice was directly encouraged by NSPs so that clients would have sufficient injecting equipment, and to limit client presentations in support of social distancing [5]. The Sydney-based data [11] may provide a further indication for the reductions in SDM use seen in our analysis, whereby the SDMs did not attract new users, while regular SDM users experienced barriers to access, such as the SDMs being out-of-stock. SDM clients may have sought out alternate sources of needles/syringes that were not the NSP, such as pharmacies or mobile delivery services. Given the documented difficulties in recruiting and surveying representative samples of clients who principally utilise SDMs [18, 19], it is uncertain how SDM clients may have responded to SDM access difficulties, or if any increase in injecting risk was experienced.

While we did not note a statistically significant decline in NSP presentations associated with the lockdowns, this was part of an overall downward trend in presentations for the MCNSP. This trend is consistent with declining NSP presentations nationally, with a 30% decline in NSP presentations between 2017 and 2021 (765,000 presentations to 525,000), a national trend that was accelerated due to the COVID-19 pandemic [5]. This decline, however, does not necessarily confer increased risk, as overall distribution of needles/syringes has increased over the same five-year period, as has needle/syringe distribution per estimated person who injects drugs [5]. These findings may suggest clients across Australia are acquiring more sterile injecting equipment in a single visit or acquiring them through SDMs. Higher levels of needle/syringe acquisition in relation to injecting frequency (individual-level coverage) have previously been associated with lower levels of injecting risk behaviours [2]. This trend may also be a strong validation of Australia’s liberal needle/syringe distribution policy.

The decline in unique SDM presentations during the lockdown periods was minimal and subsequently increased following the end of lockdowns. The four SDMs have proven to be an integral part of the MCNSP overall needle and syringe distribution service, with approximately 40% of all needles/syringes distributed via the SDMs. As a contactless method of needle/syringe acquisition, the SDMs are well placed to support the NSP during COVID-19 lockdowns, and more research is needed to understand how SDMs can be further utilised in any future crisis and how this may impact the health and well-being of clients. A greater understanding of why SDM presentations declined during lockdowns is needed to determine if this was solely due to declines in drug injection, but also to meet the changing needs of clients in these situations, particularly clients who normally prefer to access SDMs. Following prior feedback from SDM clients who reported an insufficient number of needles/syringes were distributed per SDM order, the MCNSP increased the number of needles/syringes contained in the SDM packs from six to eight. A similar evaluation may help to understand the reasons why the SDMs were potentially unpreferable to some clients during lockdowns, if this non-preference led to a decrease in needle/syringe coverage, and if amendments are needed in the event of similar, future crises.

### Limitations

Limitations to this study principally stem from the nature of the data analysed. First, the SDM data were detailed to individual SDM orders, while the available NSP data were only aggregated according to monthly counts. This represents a dissimilarity in data comparison and an unavoidable limitation. Second, the estimation of unique SDM presentations according to a 45 s cut-off is inherently arbitrary. While we presumed that 45 s was sufficient time for a person to complete an SDM order, it is possible a person may have completed multiple orders within a single unique presentation where each took longer than 45 s.

## Conclusion

In this paper, we analysed four SDMs and a managing NSP during multiple COVID-19 lockdowns in south-eastern Melbourne in 2020. While the slight, but significant decline in SDM use might suggest possible barriers to access, this may have been mitigated by SDM users acquiring needles/syringes from other sources, such as stockpiling from the NSP, as was encouraged. The decline, however, may be a concern if it led to lowered needle/syringe coverage and subsequent increase in injecting risk. Further work is needed to properly explore potential changes in needle/syringe acquisition site, and associated barriers [20]. Importantly, this work adds to the body of literature around the impacts of COVID-19 on harm reduction provision and potential areas of improvement.

## Data Availability

The datasets generated and/or analysed during the current study are not publicly available but may be available from the data custodian (david.jacka@monashhealth.org) on reasonable request.
